# The separation between the 5′-3′ ends in long RNA molecules is short and nearly constant

**DOI:** 10.1093/nar/gku1249

**Published:** 2014-11-26

**Authors:** Nehemías Leija-Martínez, Sergio Casas-Flores, Rubén D. Cadena-Nava, Joan A. Roca, José A. Mendez-Cabañas, Eduardo Gomez, Jaime Ruiz-Garcia

**Affiliations:** 1 Biological Physics Laboratory, Physics Institute, Universidad Autónoma de San Luis Potosí, Alvaro Obregon 64, San Luis Potosí, S.L.P. 78290, México; 2 División de Biología Molecular, IPICYT, Camino a la Presa San Jose s/n, San Luis Potosí, SLP 78216, México; 3 Center for Nanociences and Nanotechnology, Universidad Nacional Autónoma de México, Km. 107 Carretera Tijuana-Ensenada s/n, Baja California 22800, México; 4 Centre de Desenvolupament de Sensors, Instrumentación i Sistemes, Universitat Politecnica de Catalunya, Rambla de Sant Nebridi 10, E-0822, Terrasa, España; 5 Molecular Biophysics Laboratory, Physics Institute, Universidad Autónoma de San Luis Potosí, Alvaro Obregon 64, San Luis Potosí, SLP 78290, México

## Abstract

RNA molecules play different roles in coding, decoding and gene expression regulation. Such roles are often associated to the RNA secondary or tertiary structures. The folding dynamics lead to multiple secondary structures of long RNA molecules, since an RNA molecule might fold into multiple distinct native states. Despite an ensemble of different structures, it has been theoretically proposed that the separation between the 5′ and 3′ ends of long single-stranded RNA molecules (ssRNA) remains constant, independent of their base content and length. Here, we present the first experimental measurements of the end-to-end separation in long ssRNA molecules. To determine this separation, we use single molecule Fluorescence Resonance Energy Transfer of fluorescently end-labeled ssRNA molecules ranging from 500 to 5500 nucleotides in length, obtained from two viruses and a fungus. We found that the end-to-end separation is indeed short, within 5–9 nm. It is remarkable that the separation of the ends of all RNA molecules studied remains small and similar, despite the origin, length and differences in their secondary structure. This implies that the ssRNA molecules are ‘effectively circularized’ something that might be a general feature of RNAs, and could result in fine-tuning for translation and gene expression regulation.

## INTRODUCTION

Ribonucleic acids (RNAs) are a large family of biomolecules present in all forms of life. RNAs play central roles in coding, decoding and gene expression regulation (1,2). Moreover, some RNAs, for instance, ribozymes, have catalytic activity *per se* (3). Such roles depend on the way that the RNA molecules are structured. Disruption of the native structure at any level severely reduces the function of RNA molecules (4,5). Most functional RNA molecules exhibit a secondary structure that is highly conserved across the large evolutionary distance from bacteria to mammals, e.g. the tRNAs (6,7). Calculations of the minimum free energy secondary structures of single-stranded RNA (ssRNA) molecules indicate that the percentage of paired nucleotides (nts) (*f*) and the average duplex length (*k*) approach a constant value as the number of nts increases (8–10). This constancy for *f* and *k* has been verified for a wide range of viral and yeast ssRNA sequences (11) by application of both the mFOLD (12) and the RNA Vienna algorithms (13). Based on these findings and using the tree graph theory, statistical mechanics as well as mFOLD and the RNA subprogram from Vienna RNA Package, Yoffe *et al.* (14) proposed that the ends of RNA molecules larger than 1000 nt in length are close to each other, independent of their base composition and length. They predicted that on average, the exterior loop contour length (ℒ), the loop which contains the unpaired 5′-3′ ends, for viral RNAs was between 15 and 20 nt; for randomly computer-generated RNA sequences ℒ was around 12 nt. Using a probabilistic model and an RNA sequence of 1000 nt with pairing fraction of 0.6, Fang (15) deduced an ℒ equivalent to 14.4 nt, which was in agreement with the results of Yoffe *et al.* (14). A more rigorous mathematical treatment by Clote *et al.* (16) provided a formal confirmation of the 5′-3′ ends distance constancy and finds an ℒ value around 6 nt (depending on a stickiness parameter) for a random 1000 nt RNA sequence, while their calculations of natural RNAs showed a correlation with the molecular length. In addition, Han *et al.* (17) found a probability distribution for the 5′-3′ end distance from which the end-to-end separation of random RNA sequences ‘were distinctively lower than those reported by Yoffe *et al.*’.

Despite the inherent differences in the computed distance among theoretical methods, all of them agree that there is a short end-to-end distance of RNA molecules; therefore, if this distance is adopted *in vivo*, it could have biological relevance for their functions in the cell. The close proximity provides a so-called ‘effective circularization’ of RNA molecules that should facilitate translation (14). For instance, a small gap between the 5′-cap and the 3′ poly(A) tail promotes the interaction of the eukaryotic initiation factor eIF4E with the poly(A) binding protein (18). Likewise, in cap-independent translation, base pair (bp) complementarities between the 5′- and 3′-UTRs (5′- and 3′-untranslated regions) are essential for initiation of translation (19). Furthermore, RNA circularization has been effective in the translation of some viral RNA, including the yellow fever (20), influenza A (21), dengue (22) and viroids (23).

Even with the evident biological relevance of the end-to-end distance of RNA molecules and regardless of the theoretical calculations, no experimental measurements of this distance in long RNA molecules have yet been provided. Here, we present the first experimental determination of the end-to-end distances of RNA molecules of different biological sources, orientation and lengths (Table 1) by using single molecule Fluorescence Resonance Energy Transfer (smFRET).

**Table 1.** mRNA molecules obtained from two plant-viruses and a fungus used in this study

RNA name	Source	Length (nt)
	Monocistronic RNAs	
Anti-sense *fgen1*	*T. atroviride*	574
*fgen1*	*T. atroviride*	574
*triat1*	*T. atroviride*	1012
*chi 18–4*	*T. atroviride*	1667
*phr1*	*T. atroviride*	2012
RNA1	*BMV*	3234
RNA2	*BMV*	2865
RNA2	*CCMV*	2774
	Dicistronic RNAs	
RNA3	*BMV*	2117
RNA3	*CCMV*	2177
RNAB1B3	RNA1-RNA3 from *BMV*	5345

Details of the RNA samples can be consulted in Material and Methods and Supplementary Information S3.

## MATERIALS AND METHODS

The smFRET measurements use a modified Nikon E800 microscope. Briefly, a single-line 514-nm laser (Excelsior-515-50, Spectra-Physics) was directed through a beam expander into the back of the microscope. A 100× Plan-Apochromat objective (NA 1.4, Nikon, Japan) focused the beam down to a tight focal spot within the sample chamber. Collected back fluorescence was separated from the excitation beam by a band-pass filter 67–118 Technspec (Edmund Optics, USA) and focused into a 100 μm pinhole. The donor and acceptor fluorescence was separated by a dichroic mirror 540DRLP (Omega Optical, USA), filtered (580DF30 and 670DF40, Omega Optical) and focused into single photon counting avalanche photodiodes (SPCM-AQR-14, PerkinElmer Inc., USA). A SCB-68 card (National Instruments, USA) stores the photon rates in 1 ms bins in a computer. Photon bursts were analyzed using a Matlab (MathWorks, Natick, MA, USA) algorithm. To identify single-molecule events, we consider only bins with total photon rates above a background threshold value. FRET efficiency values were computed using the usual equation for the energy transfer efficiency $E_i = I_A^i /\left( {I_A^i + I_D^i } \right)$, where $I_A^i$ and $I_D^i$ are the background subtracted photon counts of the acceptor and donor emission, respectively, on bin *i*. The efficiencies were collected into a histogram, which was fitted with a Gaussian distribution to obtain the mean FRET efficiency and its corresponding full width at half maximum (Supplementary Figure S1a).

### smFRET calibration

The smFRET system was calibrated by determining the transfer efficiencies of fluorescently end-labeled double-stranded DNA molecules of 10, 13, 16, 19, 20, 21, 22, 25, 28 and 45 bp in length taking into account that a DNA bp has a separation of 0.34 nm. UTP-Alexa Fluor-546 and CTP-Alexa Fluor-647 (Invitrogen) were used as FRET pairs, and they were attached to DNA fragments by Klenow-dependent filling of overhanging Adenines and Guanines on each end of the DNA molecules. Thereafter, DNA fragments were purified using Sephadex G-25 gravity flow columns (GE Healthcare, USA). Calibration was performed with DNA fragments at a concentration of 90 pM in TE buffer (10 mM Tris, 1 mM ethylenediaminetetraacetic acid, pH 8.0).

### RNA isolation and cloning of the different genes

To obtain the different fungal messenger RNAs (mRNAs), *Trichoderma atroviride*, IMI 206040 strain, was grown over a sterilized cellophane sheet on potato dextrose agar (Difco) plates and incubated at 26°C for 48 h in total darkness. The mycelium was collected from the surface of the cellophane with a scalpel and immediately frozen in liquid nitrogen to prevent RNA degradation. Total RNA was isolated using Trizol® Reagent (Invitrogen, USA) according to the manufacturer's protocol. Contaminating genomic DNA was removed by DNase treatment using the TURBO RNAse-free kit (Ambion). Complementary DNA (cDNA) synthesis was performed using SuperScript II Reverse Transcriptase (Invitrogen Life Technologies), following the manufacturer's recommendations. The cDNA was quantified with a Nanodrop spectrophometer (Thermo Scientific, Wilmington, USA) and used to obtain the *fgen1* (24) and *triat1* (24) genes by polymerase chain reaction (PCR) amplifications. The *phr1* gene was amplified using cDNA obtained from *T. atroviride* (IMI 206040) mycelia exposed to a 5 min blue light pulse as described elsewhere (25). The gene *chi18-4* (26) was amplified with cDNA obtained from *T. atroviride* strain P1 (ATCC 74058) setting in plate confrontation assays against the phytopathogenic fungus *Rhizoctonia solani* as described by (27). For reverse transcriptase-PCR (RT-PCR) forward and reverse primers (Supplementary Table S3) were designed on the 5′ and 3′ UTRs. RT-PCR for *phr*-1 and *chi18-*4 was performed using GoTaq DNA polymerase (Promega) with the following conditions: 5 min at 94°C, 35 cycles of 94°C (30 s), 55°C (30 s) and 72°C (30 s), and a final extension of 72°C for 10 min. The RT-PCR for *fgen1* and *triat1* was basically as described for *phr*-1 and *chi18–4*, only changing the temperature and annealing time, to 45°C for 35 s for *fgen1*, whereas the temperature for triat1 was 44°C for 38 s. The PCR products were visually inspected by loading 4 μl onto 1% agarose gel. Each PCR product was cloned into pBlueScript SK II (+) vector using KpnI (*fgen1*) or ApaI-BamHI (*triat1*, *phr1* and *chi18-4*). *fgen1* was cloned also as antisense strand *(fgen1-).* Constructed plasmids were transformed into *Escherichia coli* strain TOP10F′ Cl_2_Ca competent cells. Plasmid DNA extraction from *E. coli* was performed by the alkaline lysis method and precipitated with ethanol. CCMV, RNA2 and RNA3 were already cloned into pMJ5 using StuI-XbaI (28), whereas BMV, RNA1, RNA2, RNA3 and RNA1+3 were already cloned into pT7T3–18U (29). Regardless of the plasmid, all genes were cloned in front of a T7 promoter. To obtain the different transcripts, all plasmids were linearized using proper restriction enzymes, and purified using the Wizard SV Clean-up system (Promega, USA). *In vitro* RNA transcription was performed using the T7 RiboMax kit as described by the manufacturer (Promega). DNA templates were removed by treatment with RNase-Free Dnase RQ1 (Promega). No 5′-cap nor 3′-Poly(A) tail were added to any of the mRNAs synthesized.

### RNA labeling

On all the RNA molecules, Alexa Fluor-546 on the 3′-end and Alexa Fluor-647 on 5′-end were used as FRET pairs. Custom made r-Adenosine-3′,5′-(bis)phosphate-8-[(6-Amino)hexyl]-amino-Alexa Fluor-546 (Jena Bioscience, Thuringia, Germany) was linked to the 3′-OH of the RNA molecule by the action of T4 RNA Ligase in 10 mM MgCl_2_, 10 mM DTT, 50 mM Tris-HCl, pH 7.8 and in the presence of 5 mM adenosine triphosphate. Labeled RNA molecules were cleaned using Amicon ultra-0.5 ml centrifugal filters, and subjected to 5′-end labeling in a three step strategy as follows: (i) RNA molecules were dephosphorylated using alkaline phosphatase, calf Intestinal (NEB), (ii) a thiophosphate group was added into the dephosphorylated 5′-end using ATPγS and T4 polynucleotide kinase (NEB), (iii) the thiol-reactive C-2 maleimide-Alexa Fluor 647 was allowed to react with the thiophosphate for 30 min at 65°C. All labeled RNA molecules were phenol extracted and precipitated using absolute ethanol, and resuspended either in TE buffer (magnesium-free condition) or TM buffer (Tris 10 mM, MgCl_2_ 5 mM, pH 8.0).

## RESULTS

**Figure 1.**
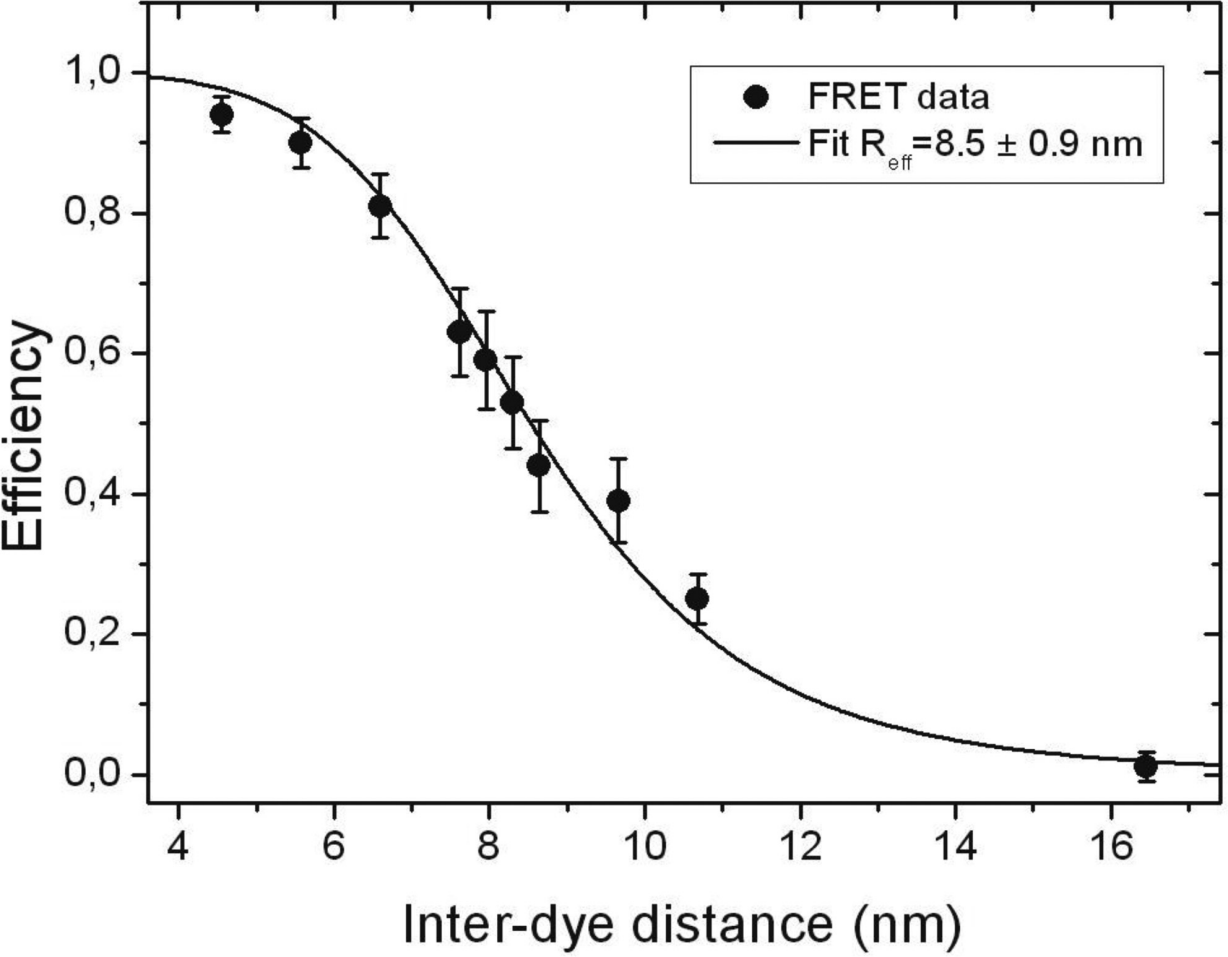
DNA calibration curve for the smFRET signals as a function of the separation of the fluorophores used as FRET pair. The error bars represent one standard deviation (±1*σ*). The solid line is the calibration curve fitted to $E^{ - 1} = 1 + (R/R_{{\rm eff}} )^6$ that gives $R_{{\rm eff}} = 8.5 \pm 0.9$ nm.

Mean FRET efficiencies were extracted from FRET histograms (Supplementary Figure S1a) and plotted as a function of the fluorophore separation. The DNA calibration curve for the smFRET signal is shown in Figure 1. We fit the data to the usual formula $E^{ - 1} = 1 + (R/R_{{\rm eff}} )^6$, where $R_{{\rm eff}} = R_0 \gamma ^{1/6}$ contains the Förster radius *R*_0_ and *γ* that depends on the quantum yields and detection efficiencies for both donor and acceptor (30). The fit to the curve gives $R_{{\rm eff}} = 8.5 \pm 0.9$ nm, where the (10%) uncertainty in the linker length of dyes used was taken into account (31).

**Figure 2.**
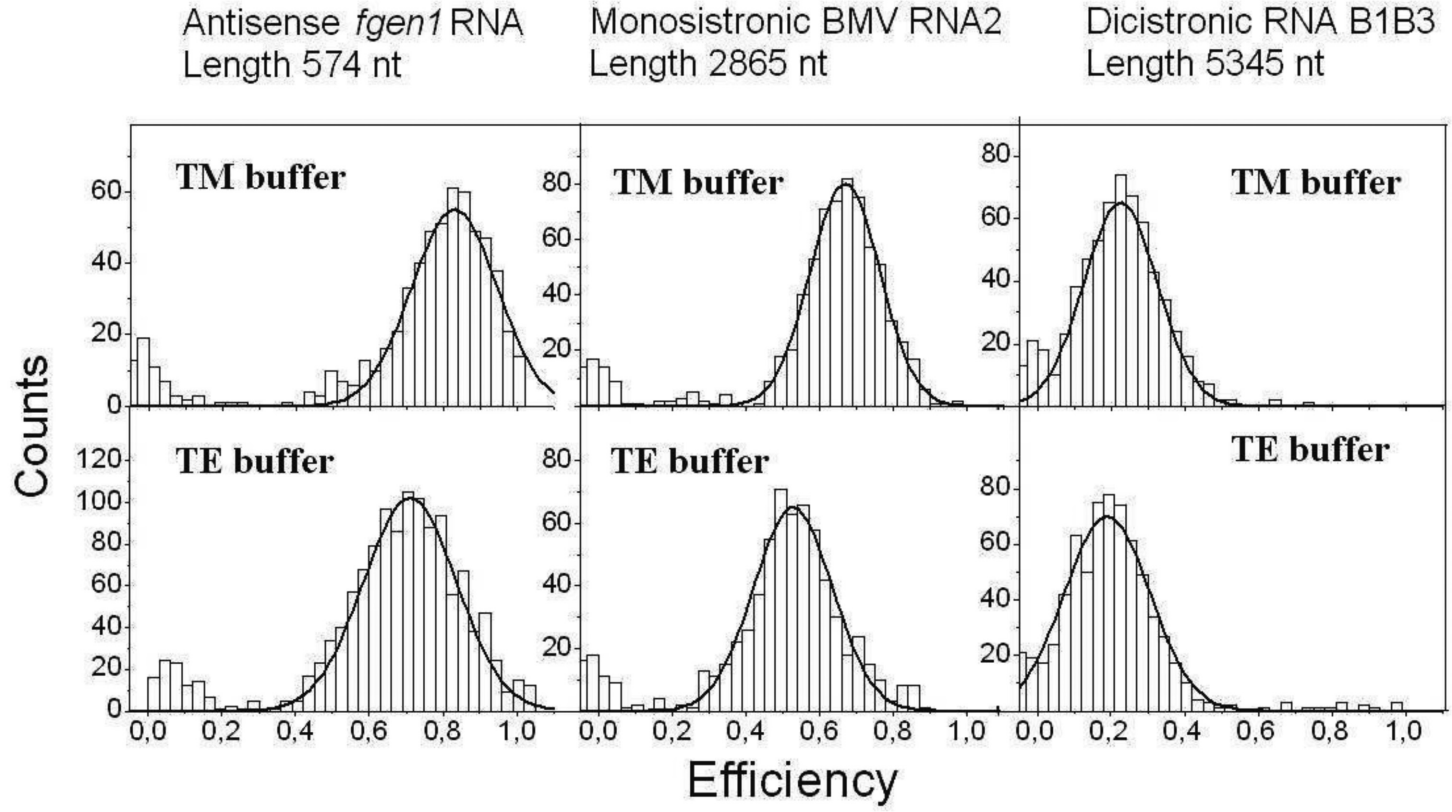
FRET histograms of three RNA molecules. Top (bottom) panel corresponds to data taken in the presence of TM (TE) buffer solution. Black lines are Gaussian fits. To avoid cluttering the rest of the RNA smFRET histograms are shown in Supplementary Figures S2a and b.

**Figure 3.**
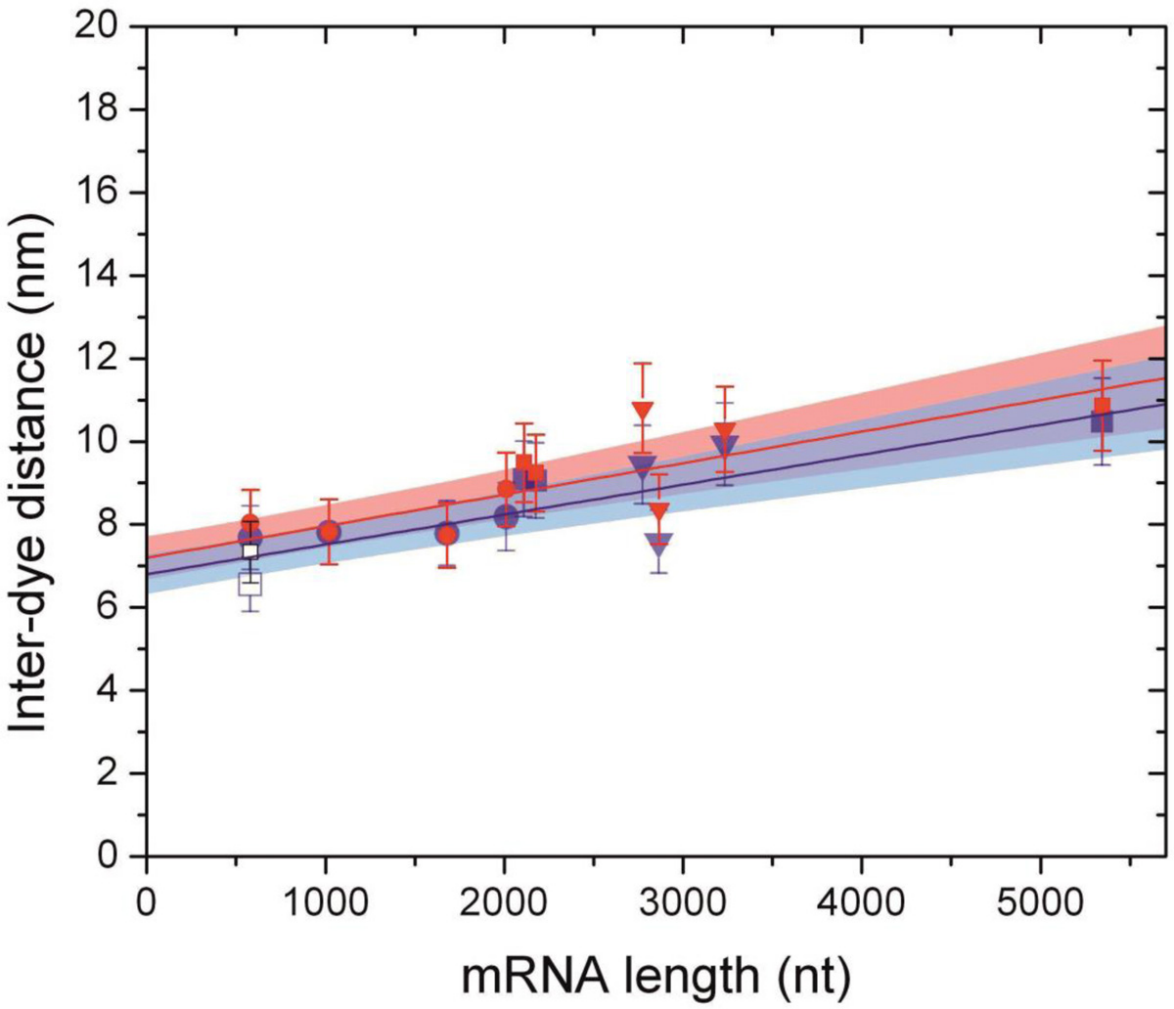
Inter-dye distances for end-labeled mRNA molecules of different lengths. Circles and triangles represent monocistronic fungal and viral mRNAs, respectively. Filled squares represent dicistronic mRNA molecules, whereas empty squares represent the antisense mRNA *fgen*1. Blue and red data are from TM and TE buffer solution smFRET experiments, respectively. Error bars correspond to ±1*σ*. The plot includes the linear fits (*y* = *a* + *bx*) with *a* = 6.8 ± 0.47 nm and *b* = 7.2 ± 2 × 10^−4^ nm/nt for TM buffer and *a* = 7.2 ± 0.5 nm and *b* = 7.6 ± 2 × 10^−4^ nm/nt for TE buffer with the 1*σ* band for each fit.

To determine the end-to-end distance we performed smFRET measurements using the 11 end-labeled mRNA molecules described in Table 1. smFRET experiments were carried out with freely diffusing mRNA molecules at 27°C either under magnesium-free conditions using TE buffer or in the presence of 5 mM magnesium using TM buffer (Figure 2 and Supplementary Figure S2a and b). As can be seen from the FRET histograms, the fluorophores are maintained around a particular separation. To determine the end-to-end separation we used the calibration curve of Figure 1. The result as a function of the mRNA length (Figure 3) shows a fluorophore separation in the range between 6.5 and 10.5 nm. The fluorophore separation is not completely constant; for example, there is a small slope of 7.2 ± 2 × 10^−4^ nm/nt in TM buffer. Increasing the RNA length by a factor of 10, as in our experiments, changes the fluorophore separation by less than 50%. Despite the important role of Mg ions on RNA tertiary stability (32), our results indicate that the end-to-end separation is not affected by tertiary interactions. Furthermore, a long ssRNA molecule might exist in a population of secondary structures (33). However, our results indicate that the end-to-end separation is not affected by the different secondary structures that an RNA molecule might adopt.

Out of the 11 RNA sequences studied, 8 RNA sequences were monocistronic, including 1 antisense complementary sequence, and 3 were dicistronic (see Table 1). They were obtained from 9 coding sequences: 4 from *T. atroviride*, 2 from CCMV and 3 from BMV. All fungal RNA sequences tested contain their UTRs, but lack the 5′-cap and Poly(A) tails. However, despite all those differences we find no significant difference in the end-to-end separation in all tested mRNAs. Although different biological roles of sense-antisense RNA have been observed and proposed (34), here we observed that the fluorophore distances of sense-antisense mRNA *fgen1* are similar (although not identical in the presence of MgCl_2_). The error bars in Figure 2 are dominated by the 10% uncertainty of the calibration. The separations in Figure 3 fluctuate within a standard deviation of 1.2 nm, which corresponds to about 14% of the measured separation.

**Figure 4.**
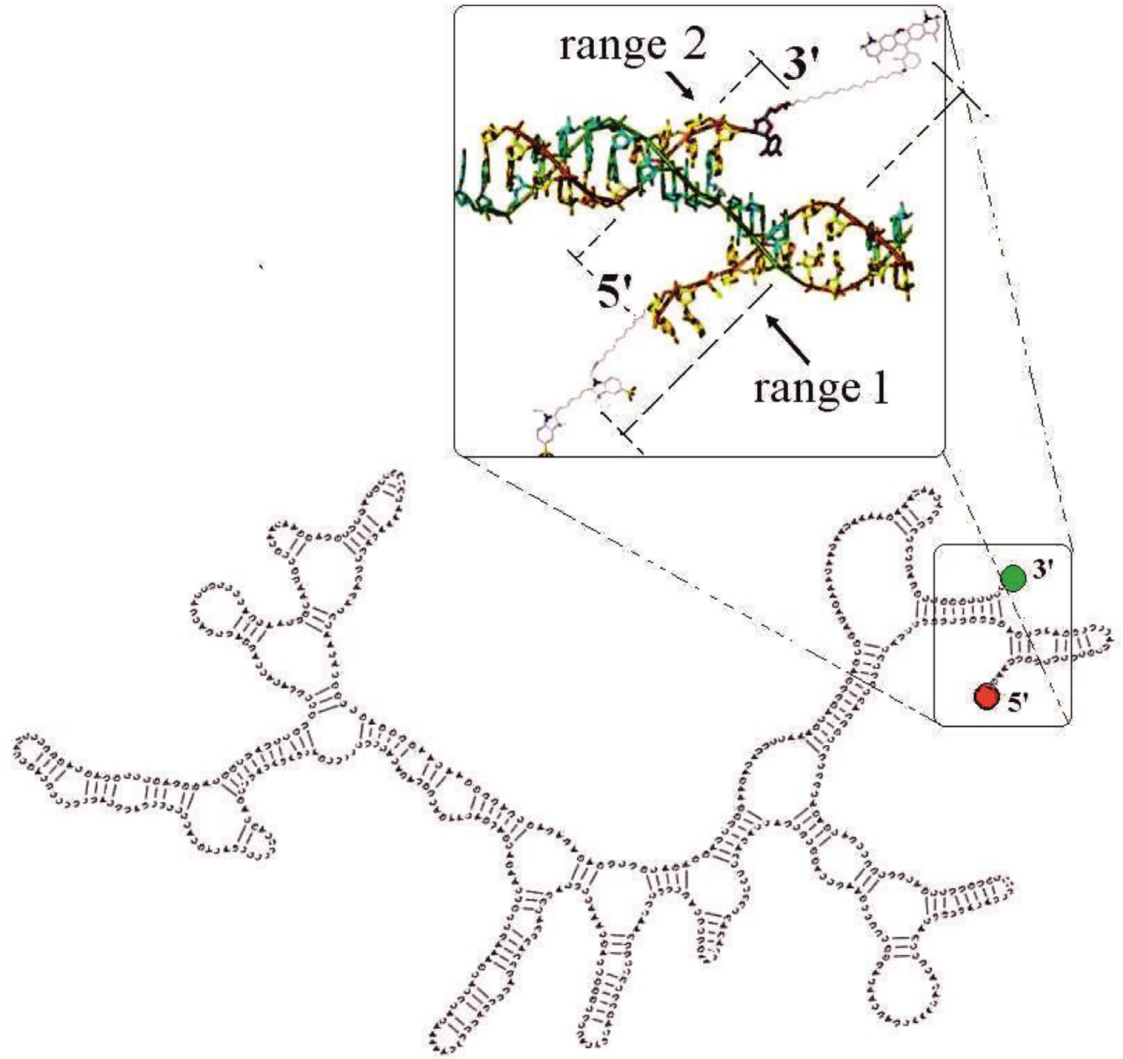
Secondary structure of mRNA *fgen1* predicted by mFOLD. The inset is a zoom of the exterior loop that contains the 5′ and 3′ ends. See text for discussion of the ranges marked in the inset.

To extract the end-to-end distance from data in Figure 3, the fluorophore linker has to be taken into account. The fluorophores attached to both ends of the short DNA molecules used for calibration point toward opposite directions adding an extra 1.5 nm to the total length. However, secondary structures of our RNA molecules predicted by mFOLD showed that in the case of the RNA molecules, fluorophores point mainly in opposite directions as well (Figure 4). smFRET histograms of the RNA molecules, are 50% wider than the limits of statistics (Supplementary Figure S2c). As in the case of DNA (Supplementary Figure S1b), fluorophore linker motion on RNA molecules contributes to the width (Supplementary Figure S2d). However, since the persistence length of ssRNA (2.1 nm at 5 mM MgCl_2_ (35)) is much smaller than that of dsDNA, there should be considerable motion of the terminals that intrinsically contributes to the end-to-end separation (considering only the exterior loop as a ssRNA). The prediction of an end-to-end distribution (36) with a persistence length of 2.1 nm gives histograms with a width greater than those we observed (Supplementary Figure S2c–e). The narrower widths measured indicate a higher rigidity of the ssRNA exterior loop (Supplementary Figure S2e). The increased rigidity may come from the fact that in most cases the exterior loop, where the ends are located, is anchored by at least two paired regions (stem-loops) on the mRNA (14) that restrict its movement.

## DISCUSSION

The separation between fluorophores we found is in the range between 6.5 and 10.5 nm (range 1). To extract the separation between 5′-3′ ends (range 2) we need to subtract from range 1 the contribution coming from the fluorophore linker lengths (Figure 4). If we consider the fluorophores as pointing out in opposite directions (with total effective linker length of 1.5 nm) we obtain an RNA end-to-end distance between 5 and 9 nm (range 2, see Figure 4). Assuming a rigid ssRNA and 0.59 nm separation between nt (37), we obtain an exterior loop contour length (*ℒ*) equivalent to 11–19 nt for range 1 and 9–16 nt for range 2. If instead we use a persistence length of 2.1 nm and the end-to-end distribution (36) we obtain an ℒ of 19–88 nt for range 1 and ℒ of 12–46 nt for range 2. We propose that range 2 is the correct one for the comparison and that the persistence length of the exterior loop should be much higher than 2.1 nm to get results consistent with the width of the histograms (Supplementary Figure S2c–e). Under these assumptions, all the measured RNA molecules give an ℒ between 9 and 16 nt, which is consistent with theoretical predictions (14–17). It is known that magnesium ions are important for the structural stability of RNA molecules (32). However, our results show that the end-to-end separation is not affected by the presence of magnesium ions. In addition, the end-to-end separation is also neither affected by the different secondary structures that an RNA molecule can adopt (33), nor the difference in origin, secondary structures and length of all tested RNAs.

The length of the majority of conserved proteins found in eukaryotes and prokaryotes species are between 70 and 1500 aminoacids (aa) in length (38–40). Therefore, the range in size of the mRNA molecules we used spans a range of what is biologically relevant. Because of the short end-to-end separation we found, our results imply that the ssRNA molecules are ‘effectively circularized’ and raise an intriguing question: is the end-to-end distance of RNAs conserved in all forms of life? If so, this structural feature must have played an important role in evolution, for example, in allowing RNA recognition to carry out their functions, a reminiscent that we can find nowadays in riboswitches, mRNA splicing (5) and transcription termination in prokaryotes (41). However, there are mRNAs that code for exceptionally large proteins, e.g. Titin that is made of ∼27 000 aa (42). Certainly, these large mRNAs are of biological significance in cell physiology; we speculate that even these large molecules also adopt similar end-to-end separation distance, since they probably use the same post-transcriptional and translational machineries.

## SUPPLEMENTARY DATA

Supplementary Data are available at NAR Online.

SUPPLEMENTARY DATA
