# Supplementary material for: The separation between the 5′-3′ ends in long RNA molecules is short and nearly constant
Source: Nucleic Acids Res. 2014 Nov 26;42(22):13963–8. doi: 10.1093/nar/gku1249 (PMC4267660; doi:10.1093/nar/gku1249)
Supplement: SUPPLEMENTARY DATA [file supp_42_22_13963__index.html]

The separation between the 5′-3′ ends in long RNA molecules is short and nearly constant — The separation between the 5′-3′ ends in long RNA molecules is short and nearly constant — SUPPLEMENTARY DATA 

# The separation between the 5′-3′ ends in long RNA molecules is short and nearly constant

## SUPPLEMENTARY DATA

**Files in this Data Supplement:**

- SUPPLEMENTARY DATA
