# Supplementary material for: The separation between the 5′-3′ ends in long RNA molecules is short and nearly constant
Source: Nucleic Acids Res. 2014 Nov 26;42(22):13963–8. doi: 10.1093/nar/gku1249 (PMC4267660; doi:10.1093/nar/gku1249)
Supplement: SUPPLEMENTARY DATA [file supp_gku1249_nar-02693-r-2014-File002.doc]

**SUPPLEMENTARY INFORMATION**

**S1. DNA FRET histograms.**

For all single-molecule FRET measurements Free-RNase sample chambers were prepared. Each sample chamber consisted of a rectangular tubing model 5005 (Vitrocom Inc.) placed on a cover glass and with an RNase free tube affixed with epoxy to each end.

Figure S1a shows the FRET efficiency histograms that were generated from photon-burst data of each DNAs constructs. The mean value and width of the FRET efficiencies were obtained by Gaussian fits.

**
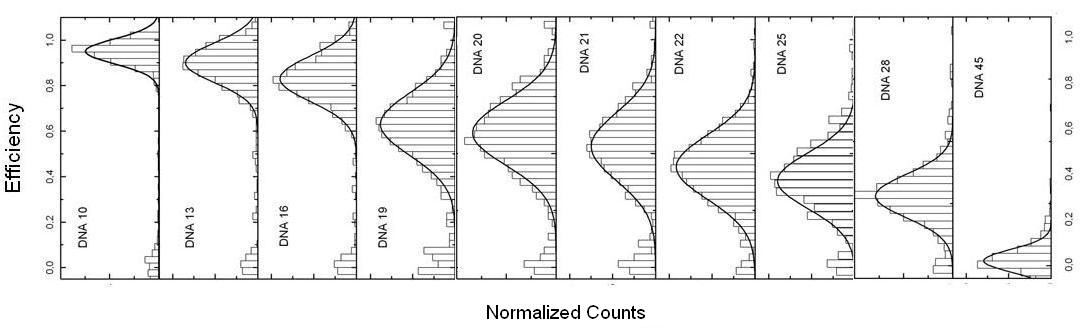
**

Figure S1a. FRET energy efficiency histograms obtained from freely diffusing single-molecule end-labeled DNA fragments of the indicated lengths (in bp). Black lines are Gaussian fits.

**Widths of smFRET histograms for the DNA segments**

The width in all histograms is not far from the one limited by statistics. In the case of the DNA

segments used for the calibration of the smFRET apparatus (Fig. S1b), we find that the increase in the

width can be well explained by considering the motion of the fluorophores in the expected range due to

the linkers. This motion contributes more in the range from medium to high efficiencies as shown in Fig. S1b.


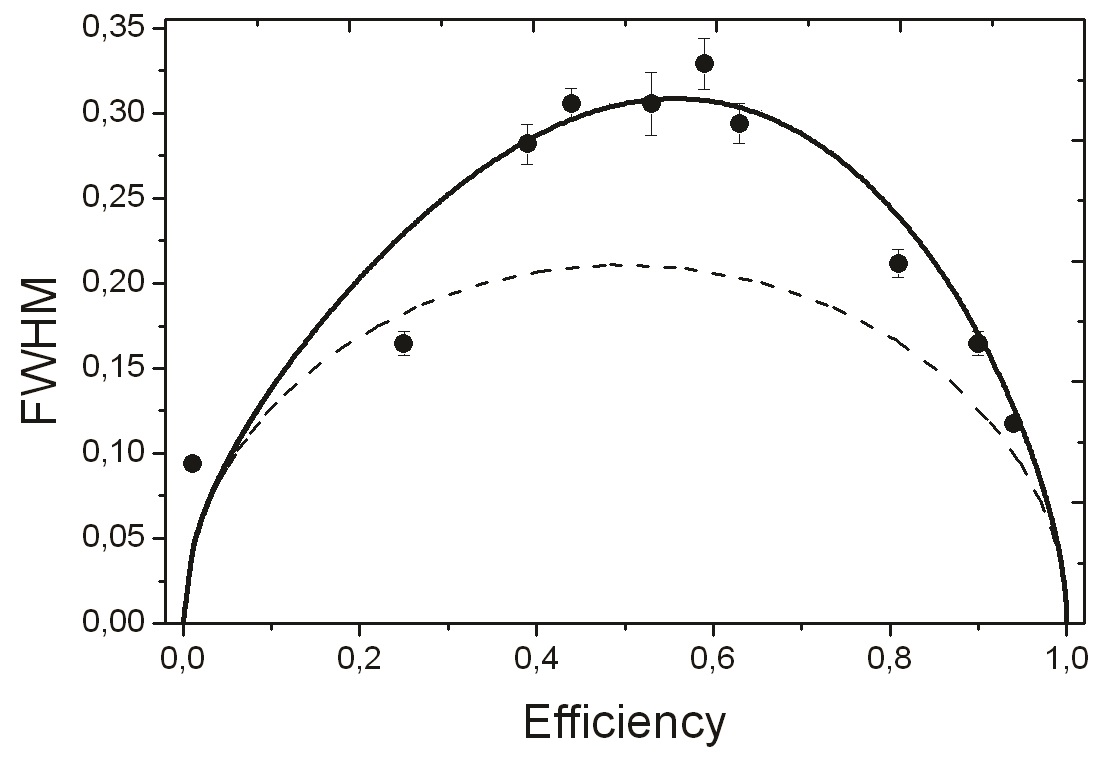


Figure S1b. Width of the smFRET DNA histograms shown in S1a as a function of the efficiency. Comparison between the width limited by statistics (dashed line) and that including also the contribution from the motion of the fluorophores within a range of 0.8 nm (black line).

**S2. RNA FRET histograms**


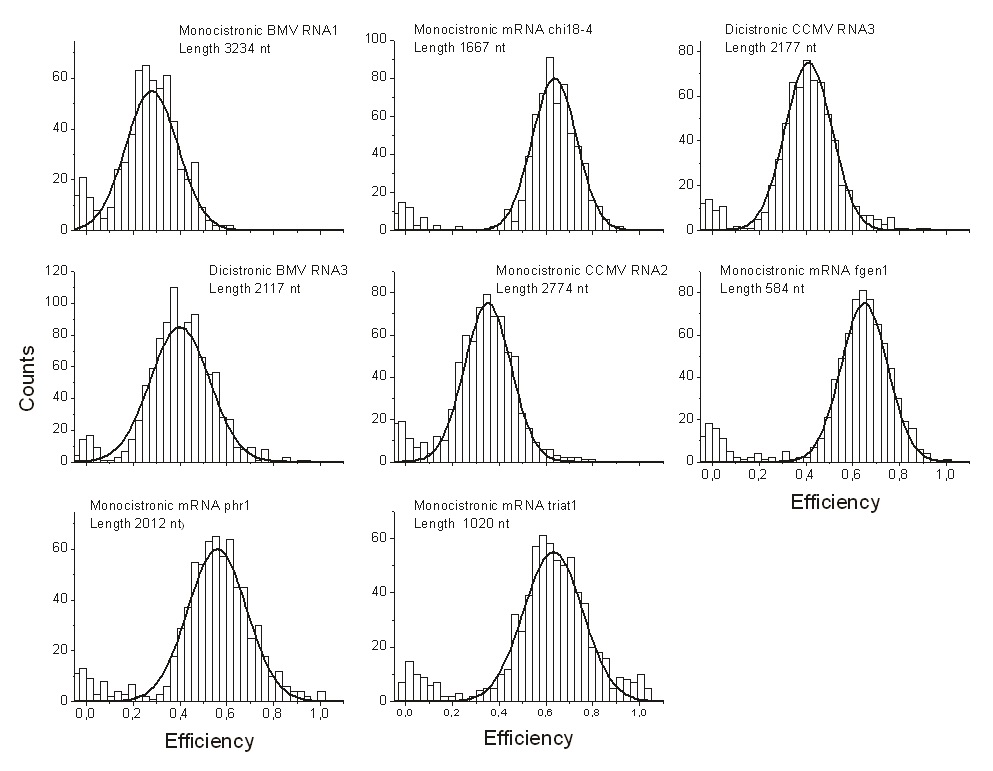


Figure S2a. Energy transfer efficiency histograms of RNA molecules obtained in TM buffer solution. Curves are Gaussian fits.


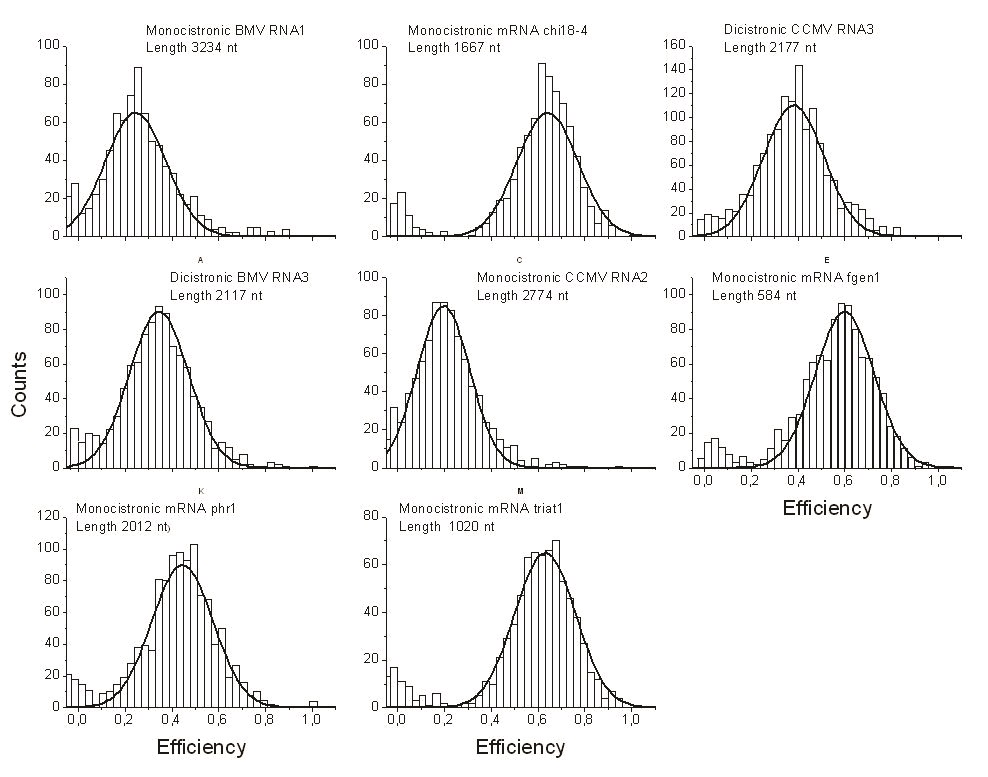


Figure S2b. Same as in Fig. S2a but with RNA samples in TE buffer solution.

**Widths of smFRET histograms for the RNA samples.**

DNA molecules have a persistence length that is larger than the DNA segments we used for the smFRET apparatus, and the end points of these segments maintain their distance. This is not the case for the ssRNA, and in an end-to-end distribution description there are variations in the end-to-end distances (considering only the exterior loop). Including these variations in the expected smFRET signal width, we get a width that is much bigger than the measured one (Fig. S2c). In order to calculate the theoretical curve, the only free parameter is the persistence length. To be able to explain the widths experimentally observed one needs to assume a bigger persistence length of the exterior loop. Figure S2d shows the expected width for a persistence length of 30 nm that gives results that are not far from the measured values. The variations in the end-to-end distribution contribute more at low efficiencies.

**
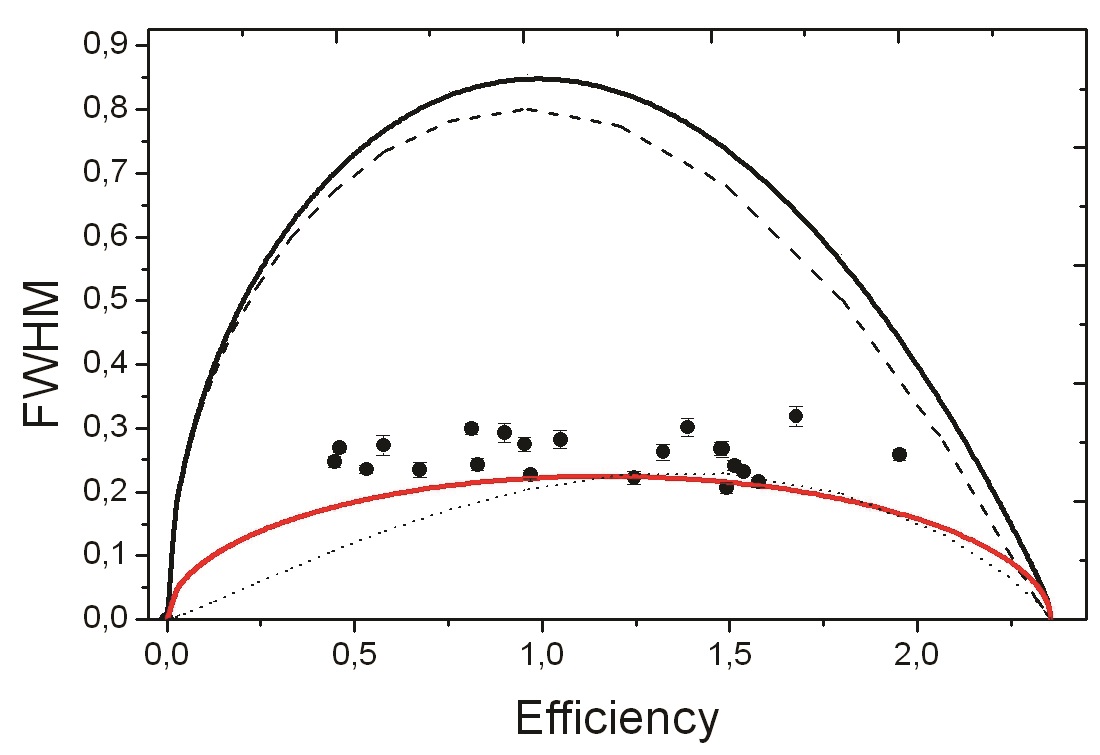
**

Figure S2c. FWHM of the RNA histograms versus efficiency (circles). Contribution to the width coming from statistics (red line), from the motion of the fluorophores within a range of 0.8 nm (dotted line), and from the variations in the distance between RNA terminals in an end-to-end distribution with a persistence length of 2.1 nm (dashed line). The black curve is the sum of all contributions.


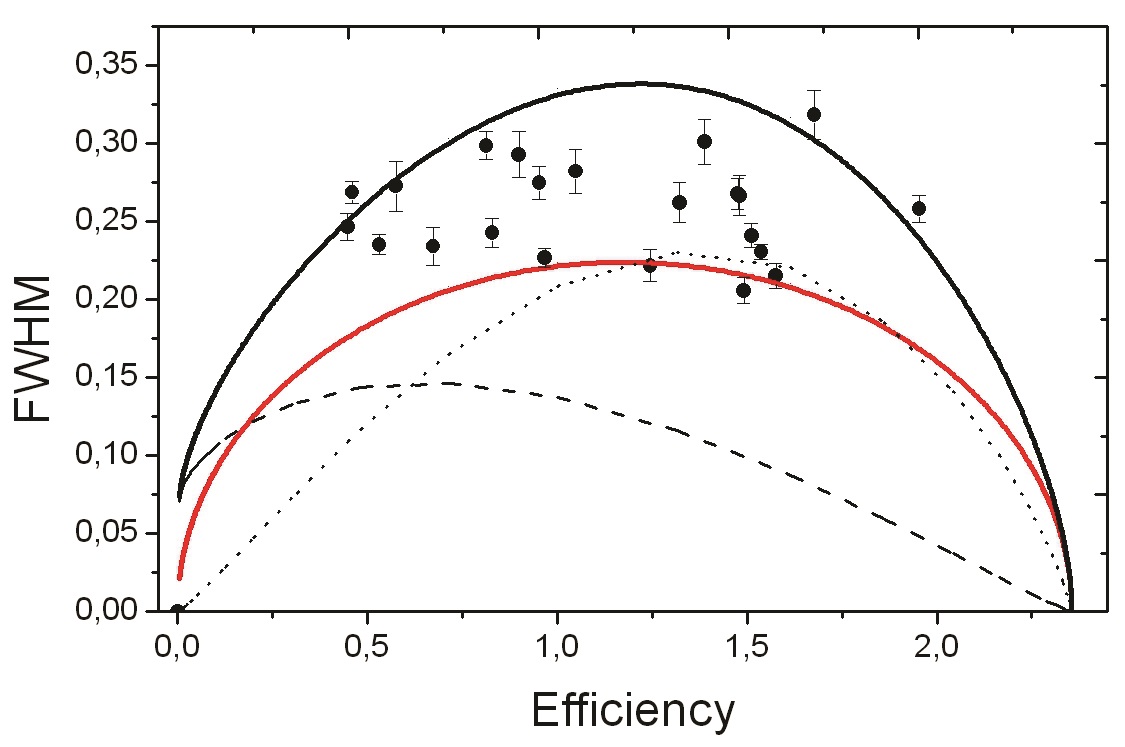


Figure S2d. Same as Fig. S2c but considering a persistence length of 30 nm for the exterior loop.


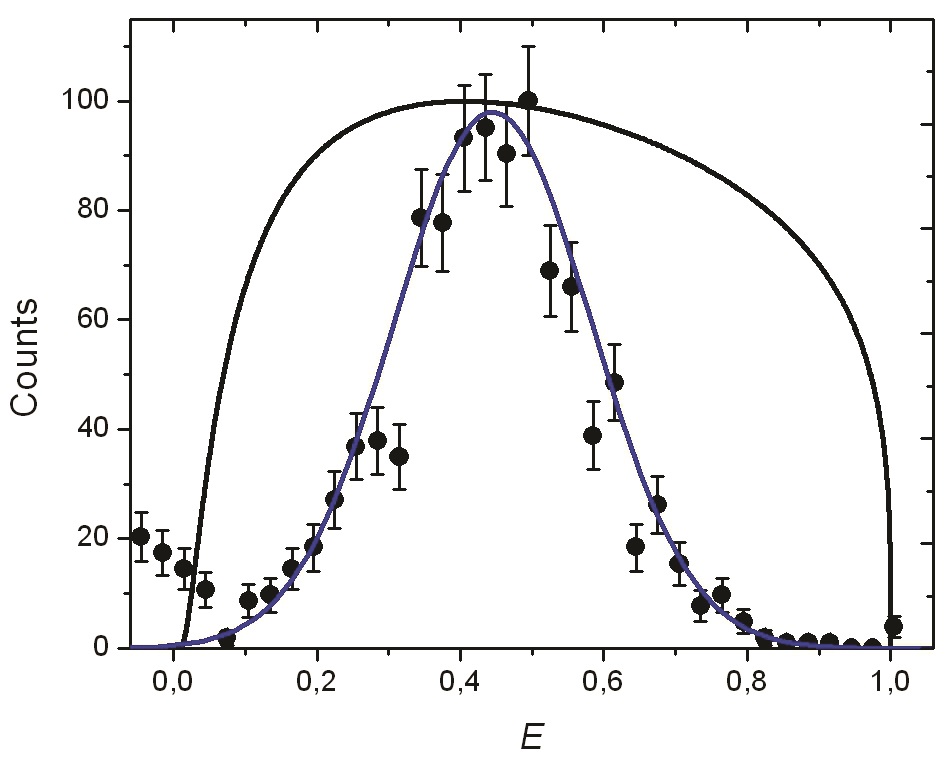


Figure S2e. Comparison between a measured smFRET histogram (circles and blue line for a Gaussian fit) and that expected from a calculated end-to-end distribution (black line). The data is from RNA *phr1* in TE buffer and we use a contour length of the exterior loop of L =25 nm so that the mean efficiency approximately corresponds to that of the data. The persistence length used here was 2.1 nm.

**S3. RNAs INFORMATION**

From the fungus *T. atroviride:* the RNA sequence of *fgen1* can be consulted with protein ID 258498 in the Department of Energy Joint Genome Institute (JGI) portal as well as the RNA sequence of RNA *triat1* but with ID 146288. The GenBank Accession Numbers for RNA sequences of *chi18-4* and *phr1* are DQ068751.1 *and* AJ009960.1, respectively. From the cowpea chlorotic mottle virus (CCMV) RNA secuences of RNA 2 and RNA3 can be consulted with the GenBank accession numbers MCCRNAA2 and HQ611268, respectively. From the brome mosaic virus (BMV): RNA1, RNA2 and RNA3 have GenBank accession numbers AB183262, NC_002027 and AB183261.1, respectively. Two additional RNA molecules were obtained with the antisense of *fgen1* and the construction of RNA1 and RNA3 of BMV virus.

**Oligonucleotide sequences for RT-PCR**

Table S3. Forward (f) and reverse (r) primers used to get four DNA genes from the filamentous fungus *T. atroviride* by polymerase chain reaction.

**
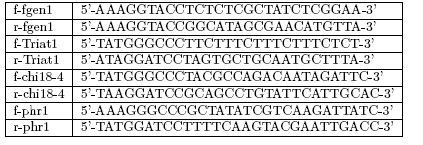
**
